# Editorial: Community series in mental illness, culture, and society: dealing with the COVID-19 pandemic, volume VI

**DOI:** 10.3389/fpsyt.2023.1233633

**Published:** 2023-06-22

**Authors:** Renato de Filippis, Samer El Hayek, Mohammadreza Shalbafan

**Affiliations:** ^1^Psychiatry Unit, Department of Health Sciences, University Magna Graecia of Catanzaro, Catanzaro, Italy; ^2^Medical Department, Erada Center for Treatment and Rehab in Dubai, Dubai, United Arab Emirates; ^3^Mental Health Research Center, Psychosocial Health Research Institute, Department of Psychiatry, School of Medicine, Iran University of Medical Sciences, Tehran, Iran

**Keywords:** coronavirus, healthcare professionals, mental health care, mental disorders, psychiatry, psychological distress, public health, SARS-CoV-2

The COVID-19 pandemic has had significant consequences, impacting not only physical health but also mental wellbeing, social interactions, and economic stability (1–3). These effects have been further shaped by individual factors and specific sociocultural dynamics, such as norms, values, and religions (4, 5). There is a need to consider, not only medical and scientific aspects, but also the broader societal and cultural dynamics when addressing public health crises (6, 7). This Research Topic explores the effects of the pandemic on mental health from the perspective of local and sociocultural factors, focusing on vulnerable and special populations, and healthcare providers.

The sixth volume of our Community Series Research Topic titled “*Mental Illness, Culture, and Society: Dealing with the COVID-19 Pandemic”* builds upon the previous five volumes (8–12) and presents nine new papers exploring how mental health is impacted by the interplay of culture and society during and after the COVID-19 pandemic.

Kuhlmann et al. argued that violence against healthcare workers (HCWs) is a serious global issue that threatens healthcare workforce retention and health system resilience, especially during the fragile post-COVID “*normalization*” period. The authors used a comparative approach, analyzing the epidemiological, political, and geographic contexts of Brazil, the United Kingdom, New Zealand, and Germany to identify similarities and differences in violence against HCWs. Overall, the results showed a general sensitivity of HCWs to violence, with women, nurses, and migrant/minority groups being particularly exposed. The authors emphasized the need for attention to this topic and to all forms of violence in the world.

The study by Huang et al. aimed to compare depressive symptoms among HCWs in high-risk areas (HRAs) and low-risk areas (LRAs) during the initial stage of the COVID-19 pandemic in China. The results showed that HCWs in LRAs had 1.96 times higher odds of having depressive symptoms than those in HRAs. There were also significant differences in workplace environment characteristics and the Health Belief Model between the two groups. The study highlighted the importance of considering the mental health of HCWs, especially in LRAs, and tailoring interventions to their specific needs.

The study conducted by Savu et al. aimed to determine the mediating role of HCWs' perception of their own health on pandemic stress, work-family conflict, work engagement, meaning and commitment to work, satisfaction of basic psychological needs, patient care, and burnout symptoms. The authors identified significant correlations between the investigated variables. In particular, HCWs with a positive perception of their own health were better at managing pandemic stress, burnout effects, and work-family imbalances.

Ayub et al. reviewed the impact of the COVID-19 pandemic on religious activities and beliefs, and explored the potential role of religious leaders and communities in mitigating the pandemic's impact through public health measures and community engagement. The authors identified the following main themes: the relationship between religious practices, beliefs, and the spread of COVID-19, and the role of religious leaders and faith communities in coping with and mitigating the impact of COVID-19. The review highlighted the essential role of religious leaders, faith-based organizations, and faith communities in promoting education, preparedness, and response efforts during the pandemic. The importance of collaboration between religious leaders, institutions, and public health officials was also emphasized.

Ryu et al. aimed to understand COVID-19 vaccine acceptance and related factors among 572 individuals with mental disorders residing in Korea. Clustering revealed three groups in relation to vaccine acceptance: totally accepting, somewhat accepting, and hesitant groups. Individuals in the high vaccine acceptance group were older, more likely to receive the influenza vaccine regularly, and more likely to trust formal information sources. The study highlighted the importance of understanding the behavioral and psychological characteristics associated with vaccine acceptance, to be able to effectively communicate its importance to individuals with mental disorders.

Carbone and Knapp investigated the use of mandatory psychiatric treatments during the COVID-19 pandemic, with a focus on the first and subsequent phases. Interviews were conducted with mental health care professionals and scholars from four countries. The analysis identified four major themes: the culture of psychiatric care services, the effect of the pandemic on involuntary hospitalizations, exceptional management of hospitalization to reduce infection spread, and policies and suggestions for more inclusive mental health treatments. The study found that during the first wave, there was a decrease in the use of involuntary treatments, while a gradual increase was observed in the following months.

With their study protocol, Qiao et al. documented the unique challenges faced by rural black women during the COVID-19 pandemic and tried to highlight their needs for effective management of social, physical, and mental health challenges. The study aimed to inform evidence-based decision-making for policymakers and to contribute to the development of public health emergency preparedness plans. This would help promote the resilience of rural Black women and their families during future infectious disease outbreaks and other public health emergencies.

The study by Park et al. examined the connections between pandemic-related factors and anxiety/depressive symptoms in young adults from South Korea and the U.S.. The findings from 1,123 participants collected during the COVID-19 lockdown period showed similar network structures in both countries, suggesting a consistent relationship between the pandemic and internalizing symptoms, irrespective of sociocultural differences. COVID-related stress and negative anticipation of the future were identified as key factors connecting pandemic-related elements to psychological distress.

Finally, Du discussed the impact of the COVID-19 pandemic on community-centered engagement and healthcare services. The author particularly described the experiences of migrant workers in Singapore and community volunteers in Shanghai and the effectiveness of coordinated community efforts in providing essential supplies and support during lockdowns Du. The article also emphasized the role of community health centers in testing and vaccination programs, especially among marginalized populations.

In brief, the articles collected in the Sixth Volume of this Research Topic provide a novel perspective on the pandemic's impact on mental health, further emphasizing the role of sociocultural, economic, and individual factors in this interplay. The influence of COVID-19 on psychiatry and mental health is significant and enduring. Further clinical and epidemiological research is necessary to address the vulnerabilities of the most fragile segments of society.

## Author contributions

All authors listed have made a substantial, direct, and intellectual contribution to the work and approved it for publication.
